# The impact of *TP53* status of tumor cells including the type and the concentration of administered ^10^B delivery agents on compound biological effectiveness in boron neutron capture therapy

**DOI:** 10.1093/jrr/rrad001

**Published:** 2023-02-10

**Authors:** Shin-ichiro Masunaga, Yu Sanada, Takushi Takata, Hiroki Tanaka, Yoshinori Sakurai, Minoru Suzuki, Mitsunori Kirihata, Koji Ono

**Affiliations:** Research Center for Boron Neutron Capture Therapy, Osaka Metropolitan University, Sakai, Osaka, 599-8531, Japan; Hanwa Daini Senboku Hospital, Sakai, Osaka 599-8271, Japan; Institute for Integrated Radiation and Nuclear Science, Kyoto University, Kumatori, Osaka 590-0458, Japan; Institute for Integrated Radiation and Nuclear Science, Kyoto University, Kumatori, Osaka 590-0458, Japan; Institute for Integrated Radiation and Nuclear Science, Kyoto University, Kumatori, Osaka 590-0458, Japan; Institute for Integrated Radiation and Nuclear Science, Kyoto University, Kumatori, Osaka 590-0458, Japan; Institute for Integrated Radiation and Nuclear Science, Kyoto University, Kumatori, Osaka 590-0458, Japan; Research Center for Boron Neutron Capture Therapy, Osaka Metropolitan University, Sakai, Osaka, 599-8531, Japan; Kansai BNCT Medical Center, Osaka Medical and Pharmaceutical University, Takatsuki, Osaka, 569-0801, Japan

**Keywords:** boron neutron capture therapy (BNCT), compound biological effectiveness (CBE) factor, TP53 status, tumor heterogeneity, quiescent (Q) tumor cell

## Abstract

Human head and neck squamous cell carcinoma cells transfected with mutant *TP53* (SAS/*mp53*) or neo vector (SAS/*neo*) were inoculated subcutaneously into left hind legs of nude mice. After the subcutaneous administration of a ^10^B-carrier, boronophenylalanine-^10^B (BPA) or sodium mercaptododecaborate-^10^B (BSH), at two separate concentrations, the ^10^B concentrations in tumors were measured using γ-ray spectrometry. The tumor-bearing mice received 5-bromo-2’-deoxyuridine (BrdU) continuously to label all intratumor proliferating (P) tumor cells, then were administered with BPA or BSH. Subsequently, the tumors were irradiated with reactor neutron beams during the time of which ^10^B concentrations were kept at levels similar to each other. Following irradiation, cells from some tumors were isolated and incubated with a cytokinesis blocker. The responses of BrdU-unlabeled quiescent (Q) and total (= *P* + Q) tumor cells were assessed based on the frequencies of micronucleation using immunofluorescence staining for BrdU. In both SAS/*neo* and SAS/*mp53* tumors, the compound biological effectiveness (CBE) values were higher in Q cells and in the use of BPA than total cells and BSH, respectively. The higher the administered concentrations were, the smaller the CBE values became, with a clearer tendency in SAS/*neo* tumors and the use of BPA than in SAS/*mp53* tumors and BSH, respectively. The values for BPA that delivers into solid tumors more dependently on uptake capacity of tumor cells than BSH became more alterable. Tumor micro-environmental heterogeneity might partially influence on the CBE value. The CBE value can be regarded as one of the indices showing the level of intratumor heterogeneity.

## INTRODUCTION

In neutron capture reaction in boron [^10^B(n,α)^7^Li], when sufficient amount of ^10^B is accumulated into the target tumor, irradiation with a sufficient number of low-energy thermal neutrons can destroy the tumor very effectively. The two particles produced in this reaction have a high linear energy transfer (LET) and release all energy within approximately one tumor cell diameter. Namely, ^10^B burden tumor cells can be killed without affecting adjacent normal cells if sufficient amount of ^10^B atoms can be selectively accumulated in the interstitial space of tumor tissue and/or intracellularly. Therefore, this tumor-selective irradiation is very likely to improve local control after boron neutron capture therapy (BNCT) [1].

The radiation doses delivered to tumor and normal tissues through BNCT are due to energy deposition from three types of ionizing radiation that differ in their LET, which is the rate of energy loss along the path of an ionizing particle: 1. low-LET γ-rays, resulting primarily from the capture of thermal neutrons by normal tissue hydrogen atoms [^1^H (n, γ) ^2^H] and contaminating γ-rays included in neutron beams employed for irradiation; 2. high-LET protons, produced by the scattering of fast neutrons and from the capture of thermal neutrons by nitrogen atoms [^14^N (n, *p*) ^14^C]; and 3. high-LET, heavier charged α particles and ^7^Li ions, released as products of the thermal neutron capture and fission reactions with ^10^B [^10^B (n, α) ^7^Li]. Since both tumor and surrounding normal tissues are always present in the radiation field, there will be an unavoidable, nonspecific background dose, consisting of both high- and low-LET radiation. However, a higher concentration of ^10^B in the tumor will result in receiving a much higher total dose than that in adjacent normal tissues, which is the basis for the therapeutic gain through BNCT [1].

To evaluate biological effects derived from only the ^10^B (n, α) ^7^Li reaction, the dose components from this reaction were determined using the concept of ‘compound biological effectiveness (CBE) factor’ [2]. The CBE factor, which is unique to both the ^10^B delivery agent and the tissue, is affected by the mode and route of administration of the ^10^B delivery agent, the ^10^B distribution in the tumor and normal tissue and more specifically the size of the nucleus in the cell and even in the target tumor cell. The CBE factor is essentially different from the classically defined RBE, which depends mainly on the quality of the delivered radiation (i.e. LET). The total radiation dose in Gy delivered to any tissue is expressed in photon-equivalent units as the sum of each of the high- and low-LET dose components multiplied by weighting factors. RBE or CBE factor is actually one of the weighting factors [2].

Tumor suppressor gene *TP53* and its protein product *p53* play a major role in the progression and suppression of cancer. It has become clear that *p53* is widely involved not only in cancer but also in the cellular response to various external factors (cellular stress) such as hypoxia, viral infection, metabolic stress, endoplasmic reticulum stress and oxidative stress [3–5] through its function as transcription factor by cell cycle arrest, DNA repair, alteration of metabolic pathway, antioxidant action, anti-angiogenesis, autophagy, aging and apoptosis. Thus, mutations in *TP53* and the resulting loss of *p53* protein function induce cancer development and progression, and decreased *p53* function is associated with cell cycle checkpoints or apoptosis and induction of angiogenesis in cancer cells. The genetic and functional status of the *TP53* gene is thought to be one of the critical factors in guiding therapeutic strategies in cancer patients.

Here, we analyzed the changes in the values of RBE for neutron-only irradiation and CBE factors for employed ^10^B delivery agents according to their concentrations when administered *in vivo*, including focusing on the dependency on *TP53* status of tumor cells using tumor cell lines with identical genetic backgrounds except for *TP53* status. The neutron capture reaction was carried out with a ^10^B delivery agent, boronophenylalanine-^10^B (BPA, C_9_H_12_^10^BNO_4_) or sodium mercaptoundecahydrododecaborate-^10^B (BSH, Na_2_^10^B_12_H_11_SH). Regarding the local tumor response, the effect not only on the total (= proliferating [P] + quiescent [Q]) tumor cell population, but also on the Q tumor cell population, was evaluated using our originally developed method for selectively detecting the response of Q tumor cells within solid tumors [6].

## MATERIALS AND METHODS

### Cells, tumors and mice

Previously established two stable transfectants, SAS/*mp53* and SAS/*neo* cells, were employed in this study. They were incubated at 37°C with Dulbecco’s Modified Eagle Medium (DMEM) containing 20 mM 2-[4-(2-hydroxyethyl)-1-) piperazinyl]ethanesulfonic acid (HEPES) and 12.5% fetal bovine serum in a conventional humidified 5% CO2 incubator. The procedures for establishment of SAS/*mp53* and SAS/*neo* cells were previously reported in detail [7–9]. Incidentally, through transfection of the plasmid pC53–248 containing the *mp53* gene (codon 248, Arg to Trp) producing the dominant negative *mp53* protein or pCMV-Neo-Bam containing the *neo* resistance marker into the human head and neck squamous cell carcinoma cell line SAS cells (JCRB (Japanese Collection of Research Bioresources) Cell Bank, Tokyo, Japan), SAS/*mp53* or SAS/*neo* cells were established, respectively. SAS/*mp53* cells express a dominant negative *p53* protein and SAS/*neo* cells have a functionally wild-type *p53* protein.

Both SAS/*neo* and SAS/*mp53* cells were harvested from exponentially growing cultures and approximately 5.0 × 10^5^ cells were inoculated subcutaneously into the left hind leg of 6–7 week old syngeneic female Balb/cA nude mice. Three weeks after inoculation, tumors approximately 7 mm in diameter were observed at each transplant site, regardless of which stable transfectant was used.

### Labeling with 5-bromo-2′-deoxyuridine (BrdU)

Two weeks after tumor cell inoculation, a mini-osmotic pump (Alzet model 2001, DURECT Corporation, Cupertino, USA) containing BrdU dissolved in physiological saline (250 mg · mL^−1^) was subcutaneously implanted to label all P tumor cells for 7 days. BrdU treatment did not change the tumor growth rate. The labeling index (LI) after continuous labeling with BrdU in SAS/*neo* and SAS/*mp53* tumor cells was 48.4% (41.7–55.1%) (mean [95% confidence limit]) and 43.2% (37–49.4%), respectively. LI reached a plateau level at these stages. Therefore, tumor cells that did not incorporate BrdU after continuous labeling for 7 days were considered Q tumor cells [6].

### ^10^B delivery agents

BPA and BSH were purchased from Katchem spol. s.r.o. (Prague, Czech Republic). Following the method reported by Coderre *et al.*, BPA was converted to fructose complex to increase its solubility [10]. Aqueous solutions of BPA were prepared at concentrations of 250 and 750 mg·kg^−1^. BSH dissolved in physiological saline (0.9%) was prepared at concentrations of 125 and 375 mg·kg^−1^. The ^10^B delivery agent solution was subcutaneously administered at the neck of tumor-bearing mice in an amount of 0.02 mL · g ^−1^ per 1 gram of mouse body weight. BPA doses of 250 and 750 mg · kg ^−1^ correspond to 12.0 and 36.0 mg ^10^B kg ^−1^, respectively. BSH doses of 125 and 375 mg·kg^−1^ are equivalent to 71.0 and 213 mg ^10^B kg^−1^, respectively.

According to our previous study [11], no overt toxicity was observed at doses below 1500 mg · kg^−1^ for BPA and below 500 mg · kg^−1^ for BSH. Based on the Certificate of Analysis and the Material Safety Data Sheet provided by the manufacturer, there was no contamination with borocapate dimer (BSSB, [^10^B_24_H_22_S_2_] ^4-^). The ^10^B concentration in tumors was measured by promt γ-ray spectroscopy using a thermal neutron guide tube installed at our reactor [12].

### Irradiation

Since the ^10^B concentration in the tumor during neutron beam irradiation is a critical determinant of the cell killing effect through BNCT, we administered ^10^B delivery agent at a selected dose of ^10^B and then started irradiation at the selected time. In order to keep the ^10^B concentration in tumors as constant as possible during irradiation, based on analysis results of preliminary ^10^B biodistribution study in tumors according to the elapsed time after administration of ^10^B delivery agent performed in advance, irradiation was started at the optimal time following 60 minutes after subcutaneous administration and completed at the optimal time by 180 minutes after subcutaneous administration. Therefore, when BPA was used and when BSH was used, irradiation was performed at different timings after administration of the ^10^B delivery agent. Administration of these ^10^B delivery agents and subsequent irradiation was carried out 21 days after tumor cell transplantation, and the transplanted tumors reached a diameter of about 7 mm.

A device made of acrylic resin and capable of holding 12 mice was used to irradiate the tumor implanted at the left hind leg of the mice. Tumor-bearing mice were fixed in place with adhesive tape and then irradiated with reactor neutron beams or γ-rays. Lithium Fluoride (LiF) thermoplastic shield was employed to avoid irradiation to body parts other than the implanted solid tumor. Neutron beam irradiation was carried out under a reactor output of 1 MW at our reactor using reactor neutron beams with a cadmium ratio of 9.4. Neutron fluence was measured from activation of gold foil on both the front and back of the tumor. Since the tumor is small and just below the surface, neutron fluence was assumed to decrease linearly from the front to back of the tumor. Therefore, we used the average neutron fluence determined from the values measured on the front and back of the irradiated tumor. Contaminated γ-ray doses including secondary γ-rays were measured with a thermoluminescent dosimeter (TLD) powder on the back of the tumor. The TLD was beryllium oxide (BeO) encapsulated in a quartz glass capsule. BeO has a fairly strong sensitivity to thermal neutrons. A thermal neutron fluence of 8 × 10^12^ cm^−2^ corresponds to a γ-ray dose of approximately 1 cGy. Details were previously reported in the following reference [13]. Eight activation foils and 14 nuclear reactions were used to estimate the neutron energy spectrum [13]. The absorbed dose was calculated using the flux-to-dose conversion factor [14]. Tumors were assumed to contain H (10.7% by weight), C (12.1%), N (2%), O (71.4%) and other elements (3.8%) [15]. The average neutron flux and kerma rate of the neutron beams were 1.0 × 10^9^ n·cm^−2^·s^−1^ and 48.0 cGy·h^−1^ in the thermal neutron range (less than 0.6 eV), 1.6 × 10^8^ n・cm^−2^・s^−1^ and 4.6 cGy·h^−1^ in the epithermal neutron range (0.6 to 10 keV) and 9.4 × 10^6^ n·cm^−2^·s^−1^ and 32.0 cGy·h^−1^ for fast neutron range (10 keV and above). The kerma rate of boron dose per Φn·cm^−2^·s^−1^ of thermal neutron flux for 1 μg·g^−1^ of ^10^B was 2.67 × 10^−8^ ΦcGy·h^−1^. The dose rate of γ-rays including contaminating γ-rays in reactor neutron beams and γ-rays due to trapping of thermal neutrons by hydrogen atoms [^1^H (n, γ) ^2^H] was 66.0 cGy·h^−1^. Each irradiation group included both BrdU-burden and unburden mice.

### Immunofluorescence staining of BrdU-labeled cells and micronucleus assay

Immediately after *in vivo* irradiation, transplanted tumors were excised from BrdU-burden mice, minced and trypsinized with PBS including 0.05% trypsin and 0.02% ethylenediaminetetraacetic acid (EDTA) at 37°C for 15 minutes. The tumor cell suspension thus obtained was incubated in a tissue culture dish containing complete medium and 1.0 μg·ml^−1^ cytochalasin B for 72 hours to allow mitosis while inhibiting cytokinesis. The cultured cells were trypsinized and fixed with ethanol. After centrifugation of the fixed cell suspension, each cell pellet was resuspended in cold Carnoy’s fixative (ethanol: acetic acid = 3: 1 volume). The suspension was then placed on a glass microscope slide and the sample dried at room temperature. The slides were treated with 2 M hydrochloric acid for 60 minutes at room temperature to dissociate histones and partially denature the DNA. The slides were then immersed in borax borate buffer (pH 8.5) to neutralize the acid. BrdU-labeled tumor cells were detected using monoclonal anti-BrdU antibody (Becton Dickinson, San Jose, Calif., USA) and fluorescein isothiocyanate (FITC) conjugated anti-mouse IgG antibody (Sigma, St. Louis, MO, USA). To observe double staining of tumor cells with green-emitting FITC and red-emitting propidium iodide (PI), cells on slides were treated with PI (2 μg·ml^−1^ in PBS) and monitored under fluorescence microscopy.

The micronucleus (MN) frequency of cells not labeled with BrdU can be determined by counting the micronuclei of binucleated cells that showed only red fluorescence. The MN frequency was defined as the ratio of the number of micronuclei to the total number of binucleated cells observed. The ratios obtained for tumors not pretreated with BrdU show MN frequencies in the total (*P* + Q) tumor cell population. Over 300 binuclear cells were counted to determine MN frequency [6].

### Clonogenic cell survival assay

Clonogenic cell survival assays were also carried out for BrdU-unburden mice using an *in vivo–in vitro* assay. Tumors were resolved by agitating for 25 minutes at 37°C in PBS containing 0.05% trypsin and 0.02% EDTA. Cell yields for SAS/*neo* and SAS/*mp53* tumors were 1.5 (1.2–1.8) × 10^7^·g^−1^ and 3.4 (2.6–4.2) × 10^6^·g^−1^, respectively. Appropriate numbers of viable tumor cells from single cell suspensions were plated in 60 or 100 mm tissue culture dishes and 16 days later colonies were fixed with ethanol, stained with Giemsa and counted. Table 1 shows the plating efficiency (PE) of the total tumor cell population and the MN frequencies of the total and Q tumor cell population for unirradiated tumors.

**Table 1:** PE and MN frequency at 0 Gy

	Total cell population		Quiescent cell population	
	SAS/*neo*	SAS/*mp53*	SAS/*neo*	SAS/*mp53*
<Plating efficiency (%)>				
^10^B-carrier (−)	65.2 ± 8.1^a^	46.5 ± 6.1		
BPA^b^ (250 mg/kg)	51.0 ± 1.9	40.4 ± 1.9		
BPA (750 mg/kg)	21.8 ± 1.7	11.5 ± 1.4		
BSH^c^ (125 mg/kg)	57.0 ± 7.0	41.5 ± 8.5		
BSH (325 mg/kg)	33.0 ± 5.1	14.3 ± 3.2		
<Micronucles frequency (x 10^−2^)>				
^10^B-carrier (−)	2.6 ± 0.4	3.0 ± 0.5	5.0 ± 1.1	7.4 ± 1.3
BPA (250 mg/kg)	4.4 ± 0.6	6.7 ± 0.9	9.2 ± 1.5	9.4 ± 1.6
BPA (750 mg/kg)	5.8 ± 0.8	7.7 ± 1.2	11.0 ± 1.5	12.0 ± 1.6
BSH (125 mg/kg)	3.6 ± 0.5	4.5 ± 0.7	9.0 ± 1.3	9.2 ± 1.4
BSH (325 mg/kg)	5.1 ± 0.8	7.4 ± 1.2	10.0 ± 1.4	11.0 ± 1.5

^a^Mean ± standard deviation (n = 6)

^b^ *L-para*-boronophenylalanine-^10^B

^c^Sodium mercaptoundecahydrododecaborate-^10^B

To confirm the stability of transfectants SAS/*neo* and SAS/*mp53*, part of the tumor cell suspensions obtained after irradiation and tumor cells from part of the colonies grown through the *in vivo*–*in vitro* assay method were subjected to Western blotting analysis for *p53* and *Bax* proteins as described in [7] and [9]. Not only the level but also the function of *p53* protein could be detected because the bax gene is a target of the *TP53* gene. As a result, it was certified that the *TP53* status of each transfectant was not changed by experimental procedures.

Two or three mice were used to evaluate each condition set and each experiment was repeated twice. To examine the differences between pairs of values, the Student’s t-test was used when the two groups could be assumed to have equal variances. Otherwise, Welch t-test was used. *P*-values are from a two-sided test. If there is a request from a reader or researcher, some of the raw data obtained in this study may be shown.

## RESULTS

Based on the data in Table 1, BPA or BSH treatment induced significantly lower PE and higher MN frequencies in both total and Q cell populations than no drug treatment (*P* < 0.05). Q cells showed a significantly higher MN frequency than the total cell population under each condition (*P* < 0.05). Moreover, although not significant, as the dose of ^10^B delivery agent increased, the changes in PE and MN frequencies tended to be more remarkable compared with no drug treatment. Furthermore, SAS/*mp53* tumors, again although not significantly, had lower PE and higher MN frequencies than SAS/*neo* tumors.

Based on the ^10^B biodistribution patterns in tumors [16], irradiation was started at 60 minutes and ended by 180 minutes at the latest after subcutaneous administration of ^10^B delivery agent. The ^10^B concentration during irradiation of tumors for BPA administration at doses of 250 and 750 mg·kg^−1^ was 22.3 ± 0.3 μg·g^−1^ and 41.7 ± 2.7 μg·g^−1^ for SAS/*neo* tumors, and 19.2 ± 2.3 μg·g^−1^ and 34.1 ± 2.3 μg·g^−1^ in SAS/*mp53* in tumors. The ^10^B concentrations during tumor irradiation for BSH administration at doses of 125 and 375 mg·kg^−1^ are 14.2 ± 0.5 μg·g^−1^ and 32.2 ± 2.6 μg·g^−1^ for SAS/*neo* tumors, and 12.7 ± 0.2 μg·g^−1^ and 28.4 ± 0.5 μg·g^−1^ in SAS/*mp53* tumors.

Data on cell survival after γ-ray irradiation only were fitted with a linear quadratic (LQ) dose relationship [17]. The cell survival curve after *in vivo* irradiation with reactor neutron beams containing both neutrons and γ-rays was called the ‘neutron beams’ cell survival curve. First, to obtain the cell survival curve for the irradiation of ‘neutrons only’ excluding γ-rays, the data on the cell survival after irradiation with the reactor neutron beams were normalized with the data on cell survival for γ-rays only by dividing the data for neutron beams by the data for γ-rays only [2, 17]. At this normalization, the dose reduction factor (DRF) of 0.45 for γ-ray irradiation was employed because the neutron beams contained γ-rays with dose rates well below 1 Gy·min^−1^, that is, 0.011 Gy·min^−1^ [2]. Next, the data on cell survival when ‘neutrons only’ was irradiated after no administration of ^10^B delivery agent was fitted to the LQ dose relationship. Second, the data on cell survival for irradiation with ‘neutrons only’ after ^10^B-carrier administration were normalized with the data on cell survival for irradiation with ‘neutrons only’ without ^10^B-carrier by dividing the data for ‘neutrons only’ with ^10^B-carrier by the data for ‘neutrons only’ without ^10^B-carrier. Then, the data on cell survival for irradiation at the ‘^10^B dose’ derived only from ^10^B (n, α) ^7^Li reaction were determined. This ‘^10^B dose’ is the physically absorbed dose truly originating from high LET, heavier-charged α particles and ^7^Li ions released as products only from the thermal neutron capture and fission reactions with ^10^B [^10^B (n, α)^7^Li] only.

With the ^10^B-carrier, the surviving fraction (SF) and MN frequency were lower and higher than without the ^10^B-carrier respectively due to the slight genotoxicity of the ^10^B-carrier, even without irradiation (Table 1), as shown in our previous reports [11, 16, 18]. Therefore, the net MN frequency was used for background correction to rule out the genotoxic effects of the ^10^B-carrier itself. Net MN frequency is the MN frequency for irradiated tumors minus the MN frequency for unirradiated tumors.

Data on the net MN frequency after γ-ray irradiation alone were also fitted to the LQ dose relationship [17]. First, to obtain the data on the net MN frequency of irradiation of ‘neutrons only’ excluding γ-rays, the data on the net MN frequency after irradiation with the reactor neutron beams is normalized with the data for γ-ray irradiation only. The data for γ-rays only was subtracted from those for ‘neutron beams.’ The DRF of 0.45 for γ rays was again used at this normalization. Next, the data on the net MN frequency for ‘neutrons only’ irradiation after no administration of ^10^B-carrier was fitted to the LQ dose relationship. Second, the data on the net MN frequency for irradiation with ‘neutrons only’ with ^10^B-carrier were normalized with the data for ‘neutrons only’ without ^10^B-carrier by subtracting the data for ‘neutrons only’ without ^10^B-carrier from the data for ‘neutrons only’ with ^10^B-carrier. Then, the data on the net MN frequency for irradiation at the ‘^10^B dose’ derived only from ^10^B (n, α) ^7^Li reaction were determined [2, 19].

**Fig. 1:**
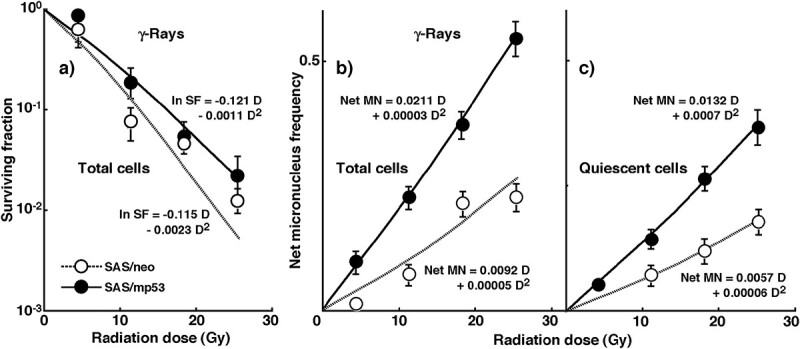
**a)** shows cell survival curves in total tumor cells, and **b)** & **c)** show the net MN frequencies in total & Q tumor cells, respectively, after *in vivo* irradiation with γ-rays only, as a function of the physical radiation dose in SAS/*neo* (open symbols) and SAS/*mp53* (solid symbols) tumor cells. SF; surviving fraction, D; physical radiation dose. Bars represent standard errors (*n* = 6).

Figure 1(a–c) shows the cell survival curves and net MN frequencies of total and Q tumor cells, respectively, after *in vivo* irradiation with γ-rays only. The SF of SAS/*mp53* tumor cells was significantly higher than that of SAS/*neo* tumor cells (*P* < 0.05). SF was expressed as a function of ln SF = − 0.115 D - 0.0023 D^2^ [D: radiation dose (Gy)] for SAS/*neo* tumors, and was ln SF = − 0.121 D - 0.0011 D^2^ for SAS/*mp53* tumors. The net MN frequencies of SAS/*neo* tumor cells and Q cells were significantly lower than those of SAS/*mp53* tumor cells and total tumor cells (*P* < 0.05), respectively. The net MN frequencies of total and Q tumor cells from SAS/*neo* tumors were expressed as a function of net MN fr_T_ = 0.0092 D + 0.00005 D^2^ and net MN fr_Q_ = 0.0057 D + 0.00006 D^2^, respectively. Those of total and Q tumor cells from SAS/*mp53* tumors were expressed as a function of net MN fr_T_ = 0.0211 D + 0.00003 D^2^ and net MN fr_Q_ = 0.0132 D + 0.0007 D^2^, respectively.

**Fig. 2:**
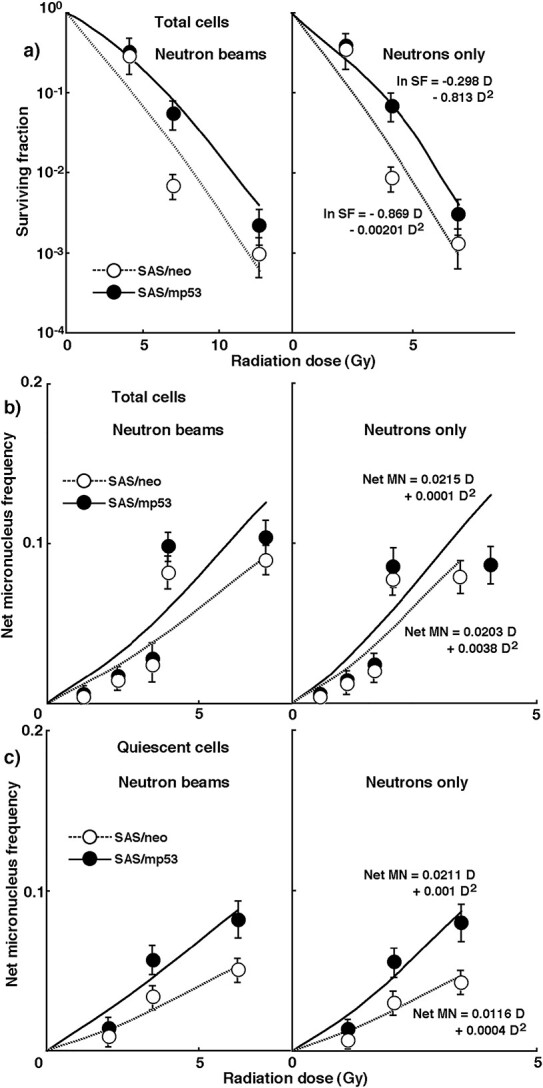
**a)** shows cell survival curves in total tumor cells, and **b)** & **c)** show the net MN frequencies in total & Q tumor cells, respectively, after *in vivo* irradiation using neutron beams without the ^10^B-carrier as a function of the physical radiation dose in SAS/*neo* (open symbols) and SAS/*mp53* (solid symbols) tumor cells. SF; surviving fraction, D; physical radiation dose. Bars represent standard errors (n = 6).

Figure 2(a–c) shows cell survival curves and net MN frequencies of total and Q tumor cells after *in vivo* irradiation with neutron beams without ^10^B-carriers, respectively. To obtain data on cell viability and net MN frequency of ‘neutrons only’ without ^10^B-carrier, the data of ‘neutron beams’ without ^10^B carrier was normalized with the above data for irradiation with γ-rays only. The SFs of ‘neutrons only’ without ^10^B-carrier were expressed as a function of ln SF = −0.869 D -0.0201 D^2^ and ln SF = −0.298 D - 0.813 D^2^ for SAS/*neo* and SAS/*mp53* tumor cells, respectively. The ‘neutrons only’ net MN frequency of SAS/*neo* tumor cells without ^10^B carriers in total and Q tumor cells was a function of net MN fr_T_ = 0.0203 D + 0.0038 D^2^ and net MN fr_Q_ = 0.0116 D + 0.0004 D^2^, respectively. Those of total and Q tumor cells in SAS/*mp53* tumor cells were expressed as a function of net MN fr_T_ = 0.0215 D + 0.0001 D^2^ and net MN fr_Q_ = 0.0211 D + 0.001 D^2^, respectively. The slope of each curve became steeper in the order of ‘neutron beams’ < ‘neutrons only.’ This is probably because the proportion of high LET radiation components increased in this order. Although the sensitivity of Q cells was still lower than that of total cells, neutron beam irradiation reduced the difference in radio-sensitivity between total and Q cells compared to irradiation with γ-rays only. Furthermore, neutron beam irradiation reduced the difference in radio-sensitivity between SAS/*neo* and SAS/*mp53* tumor cells in both total and Q tumor cells.

**Fig. 3:**
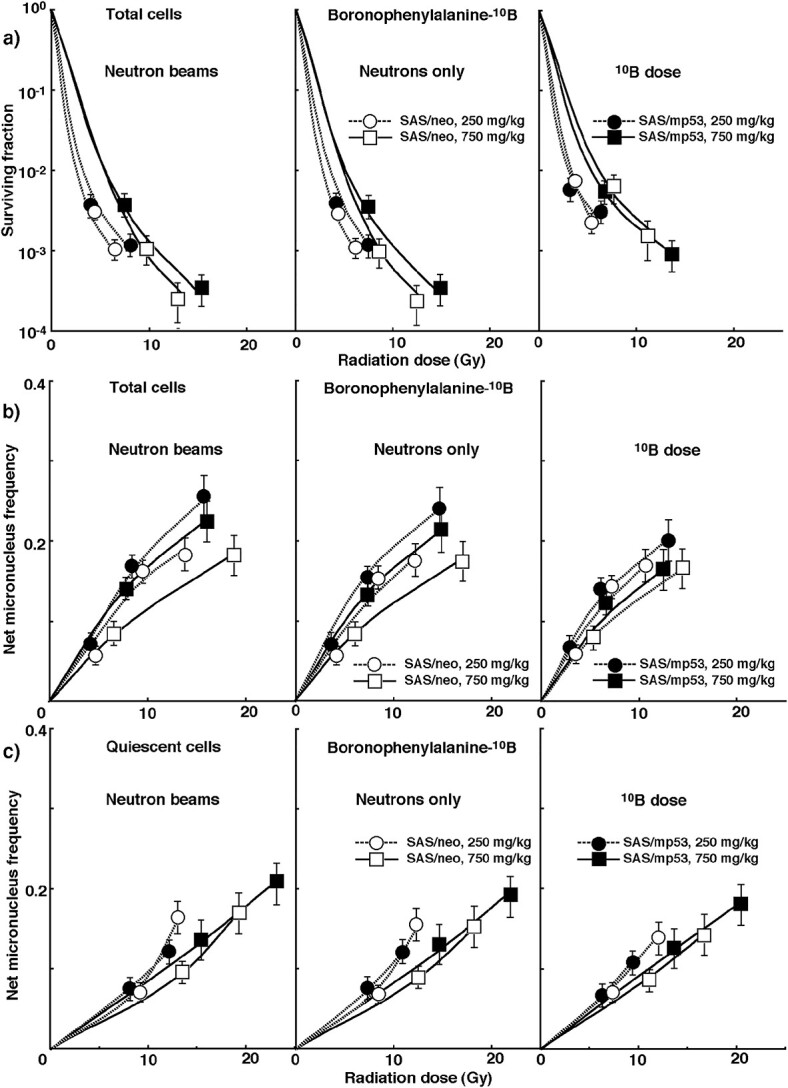
**a)** shows cell survival curves in total tumor cells, and **b)** & **c)** show the net MN frequencies in total & Q tumor cells, respectively, after *in vivo* irradiation using neutron beams following administration of boronophenylalanine-^10^B at the dose of 250 mg·kg^−1^ (circles) or 750 mg·kg^−1^ (squares) as a function of the physical radiation dose in SAS/*neo* (open symbols) and SAS/*mp53* (solid symbols)) tumor cells. The data for irradiation with reactor ‘neutron beams,’ with ‘neutrons only’ and at the ‘^10^B dose’ are shown in the left, central and right panels, respectively. Bars represent standard errors (*n* = 6).

**Fig. 4:**
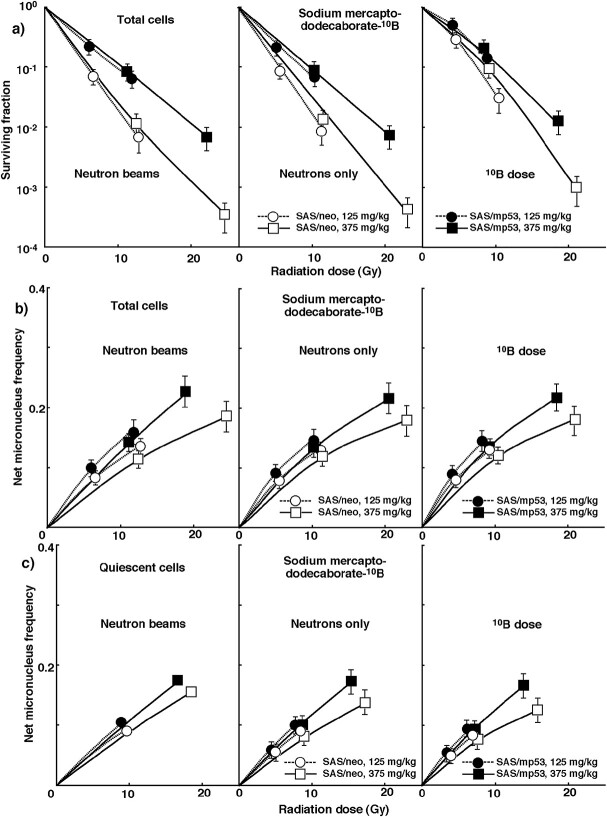
**a)** shows cell survival curves in total tumor cells, and **b)** & **c)** show the net MN frequencies in total & Q tumor cells, respectively, after *in vivo* irradiation using neutron beams following administration of sodium mercaptododecaborate-^10^B at the dose of 125 mg·kg^−1^ (circles) or 375 mg·kg^−1^ (squares) as a function of the physical radiation dose in SAS/*neo* (open symbols) and SAS/*mp53* (solid symbols) tumor cells. The data for irradiation with reactor ‘neutron beams,’ with ‘neutrons only’ and at the ‘^10^B dose’ are shown in the left, central and right panels, respectively. Bars represent standard errors (n = 6).

To obtain irradiation data at ‘^10^B dose’ derived only from ^10^B (n, α) ^7^Li reaction, the net MN frequency data for ‘neutron beam’ irradiation with ^10^B carriers were normalized with γ-ray only irradiation data and further with ‘neutron only’ irradiation data without ^10^B-carrier. Figure 3(a–c) shows the cell survival curve and the net MN frequency of total and Q tumor cells, respectively, as a function of physically absorbed radiation dose after *in vivo* irradiation after BPA administration. Figure 4(a–c) shows cell survival curves and net MN frequency in total and Q tumor cells, respectively, as a function of physically absorbed radiation dose after *in vivo* irradiation after BSH administration. In Figs 3 and 4, irradiation of ‘neutron beams’ including γ-rays, ‘neutrons only’ excluding contribution of γ-rays and ‘^10^B dose’ further excluding contribution of neutron irradiation in the absence of ^10^B are shown in the left, center and right panels respectively. The slope of each curve became steeper in the order of ‘neutron beams’ < ‘neutrons only’ < ‘^10^B dose.’ This is probably due to the increasing proportion of high LET radiation components in this order. Furthermore, in this order, the difference in radio-sensitivity between total and Q tumor cells and between SAS/*neo* and SAS/*mp53* tumor cells tended to decrease.

The data represented on each graph fitted with a LQ dose relationship are those for which equations for regression curves are shown on the graphs. In other words, the LQ dose relationship was used to analyze the data after γ-ray only irradiation (Fig. 1(a–c) and the data after neutrons only irradiation, excluding mixed *γ*-ray components (Right panels of Fig. 2(a–c). For data other than these data, (Left panels of Fig. 2(a–c), All panels of Figs 3 and 4), the curves were just shown that were thought to be the best fit after displaying the data on graphs. Therefore, it is unclear whether these curves should be fitted with the LQ dose relationship or not.

The data in Figs 1–4 are used to evaluate the RBE in ‘neutrons only’ without ^10^B-carriers and the CBE factors for BPA and BSH in both total and Q tumor cells compared with γ-rays. (Table 2). This value is calculated using the dose required to obtain each endpoint using γ-rays only and the dose of ‘neutrons only’ without ^10^B-carrier or ‘^10^B dose’ with ^10^B-carrier. The higher the number, the greater the biological effect compared with γ-rays. Overall, the value of Q cells was significantly higher than that of total cells (*P* < 0.05). BPA showed higher and lower CBE factors than BSH in the total and Q tumor cells, respectively. Incidentally, based on the fact that SAS/*mp53* tumor cells are more radio-resistant to γ-rays in terms of the SF than SAS/*neo* tumor cells (Fig. 1), the RBE and CBE values of SAS/*mp53* tumor cells were larger than those for SAS/*neo* tumor cells. However, SAS/*mp53* tumor cells showed smaller RBE and CBE values in terms of net MN frequency due to the fact that SAS/*mp53* tumor cells induced higher net MN frequency values than SAS/*neo* tumor cells after γ-ray irradiation (Fig. 1).

**Table 2:** Values of RBE^a^ and CBE^b^ factors

	Total cell population		Quiescent cell population	
	SAS/*neo*	SAS/*mp53*	SAS/*neo*	SAS/*mp53*
<Surviving fraction = 0.03>				
^10^B-carrier (−)	4.7	5.0		
BPA^c^ (250 mg/kg)	8.0	11.0		
BPA (750 mg/kg)	4.3	6.5		
BSH^d^ (125 mg/kg)	1.7	1.8		
BSH (325 mg/kg)	1.4	1.5		
<Net micronucles frequency = 0.1>				
^10^B-carrier (−)	2.5	1.5	2.8	1.9
BPA (250 mg/kg)	1.8	1.5	2.0	1.6
BPA (750 mg/kg)	1.5	1.3	1.6	1.4
BSH (125 mg/kg)	1.7	1.4	2.2	1.7
BSH (325 mg/kg)	1.3	1.2	1.7	1.5

^a^RBE (relative biological effectiveness) is the ratio of the dose of radiation dose necessary to obtain each end-point using γ-rays only to that needed to obtain each end-point using ‘neutrons only’ dose without ^10^B-carrier administration.

^b^CBE (compound biological effectiveness) is the ratio of the dose of radiation dose necessary to obtain each end-point using γ-rays only to that needed to obtain each end-point using the ^10^B dose with ^10^B-carrier administration.

^c^ *L-para*-boronophenylalanine-^10^B

^d^Sodium mercaptoundecahydrododecaborate-^10^B

To examine the difference in radio-sensitivity between total and Q tumor cells, the dose modifying factor, that is, the ratio of the radiation dose required to obtain each net MN frequency in the Q cells to the radiation dose required to obtain each net MN frequency in total cells was calculated using the data in Figs 1–4 (Table 3). The higher the number, the greater the difference in radio-sensitivity between total and Q tumor cells. All values were significantly greater than 1.0. Overall, SAS/*neo* tumor cells showed greater values than SAS/*mp53* tumor cells. The use of neutron beams alone without the ^10^B-carrier reduced the difference in radio-sensitivity under γ-ray irradiation. Regarding both SAS/*neo* and SAS/*mp53* tumor cells, the differences in radio-sensitivity were reduced in the following order: γ-rays only>^10^B dose with BPA > ^10^B dose with BSH > Neutrons only without ^10^B-carrier.

**Table 3:** Dose modifying factors^a^ for Q tumor cells relative to the total tumor cell population

^10^B-carrier	SAS/*neo*	SAS/*mp53*
<Net micronucleus frequency = 0.1>		
γ-Rays only	1.6 ± 0.15^b^	1.5 ± 0.15
Neutrons only	1.3 ± 0.2	1.2 ± 0.1
BPA^c^ (250 mg/kg)	1.9 ± 0.2	1.6 ± 0.2
BPA (750 mg/kg)	2.0 ± 0.2	1.7 ± 0.2
BSH^d^ (125 mg/kg)	1.4 ± 0.15	1.3 ± 0.15
BSH (375 mg/kg)	1.5 ± 0.1	1.4 ± 0.1

^a^The ratio of the dose of radiation necessary to obtain each end-point without a ^10^B-carrier to that needed to obtain each end-point with a ^10^B-carrier.

^b^Mean ± standard deviation (*n* = 6)

^c^ *L-para*-boronophenylalanine-^10^B

^d^Sodium mercaptoundecahydrododecaborate-^10^B

To examine the difference in radio-sensitivity between SAS/*neo* and SAS/*mp53* tumor cells, the dose-modifying factor (DMF) based on the difference in *TP53* status of tumor cells was calculated using the data in Figs 1–4 (Table 4). In terms of the SFs, SAS/*mp53* tumor cells showed more radio-resistant to γ-rays than SAS/*neo* tumor cells (Fig. 1). The value of the DMF was the ratio of the radiation dose required to obtain SF = 0.03 for SAS/*mp53* tumor cells to the radiation dose required to obtain SF = 0.03 for SAS/*neo* tumor cells. The values of the DMFs for ‘neutrons only’ dose without ^10^B-carrier were smaller than those after γ-ray irradiation only. When a ^10^B-carrier was used, the values were larger than without a ^10^B-carrier, especially in the use of BPA than BSH with smaller rather than larger administered doses of a ^10^B-carrier. In terms of the net MN frequency, SAS/*mp53* tumor cells showed higher frequencies than SAS/*neo* tumor cells after γ-ray irradiation (Fig. 1). The value of the DMF was the ratio of the radiation dose required to obtain net MN frequency = 0.1 in SAS/*neo* tumor cells to the radiation dose required to obtain net MN frequency = 0.1 in SAS/*mp53* tumor cells. Overall, the values in total tumor cells were larger than Q tumor cells. Again, the values of the DMFs after irradiation with ‘neutrons only’ without ^10^B-carrier were smaller than those after γ-ray irradiation only, and were still larger than 1.0. When a ^10^B-carrier was used, the values of the DMFs were further smaller than without a ^10^B-carrier, especially in the use of BPA than BSH with smaller rather than larger administered doses of a ^10^B-carrier, but were still larger than 1.0, meaning that SAS/*mp53* still showed higher net MN frequencies than SAS/*neo* tumor cells.

**Table 4:** DMFs^a,b^ based on the difference in *TP53* status of SAS tumor cells

	Surviving fraction = 0.03^a^		Net micronucleus frequency = 0.1^b^
	Total cells	Total cells	Quiescent cells
γ-rays	1.5 ± 0.2^c^	2.35 ± 0.3	2.1 ± 0.2
^10^B-carrier (−)	1.3 ± 0.1	1.5 ± 0.2	1.35 ± 0.1
BPA^d^ (250 mg/kg)	1.45 ± 0.2	1.25 ± 0.1	1.2 ± 0.1
BPA (750 mg/kg)	1.4 ± 0.2	1.3 ± 0.1	1.25 ± 0.1
BSH^e^ (125 mg/kg)	1.4 ± 0.2	1.3 ± 0.1	1.25 ± 0.1
BSH (325 mg/kg)	1.35 ± 0.1	1.4 ± 0.2	1.3 ± 0.1

^a^The ratio of the dose of radiation necessary to obtain 0.03 of the SF in SAS/mp53 tumor cells to that needed to obtain 0.03 of the SF in SAS/neo tumor cells.

^b^The ratio of the dose of radiation necessary to obtain 0.1 of the net MN frequency in SAS/neo tumor cells to that needed to obtain 0.1 of the net MN frequency in SAS/mp53 tumor cells.

^c^Mean ± standard deviation (*n* = 6)

^d^ *L-para*-boronophenylalanine-^10^B

^e^Sodium mercaptoundecahydrododecaborate-^10^B

## DISCUSSION

^10^B delivery agents were given subcutaneously rather than intraperitoneally, so that higher concentrations of ^10^B in tumors could be maintained longer. In both SAS/*neo* and SAS/*mp53* tumors, the distribution pattern of ^10^B from BPA administered subcutaneously at doses of 250 and 750 mg·kg^−1^ was similar to the distribution from BSH at doses of 125 and 375 mg·kg^−1^, respectively, except at 30 minutes after administration when the concentration of ^10^B from BSH was higher than that from BPA [16]. The ^10^B concentration in BPA-treated tumors during irradiation starting 60 minutes and ending by at latest 180 minutes after administration was similar to BSH-treated tumors [16].

Solid tumors, especially human tumors, are thought to contain a higher proportion of Q cells [6, 20]. The presence of Q tumor cells, probably in part due to hypoxia and depletion of nutrients in the tumor core, is consequence of the lack of vascular supply [20, 21]. This induces MN formation in Q tumor cells even at 0 Gy (Table 1) and may induce higher levels of tumor heterogeneity [20, 21]. Q tumor cells were shown to be significantly less radio-sensitive than total tumor cells (Fig. 1). This means that more Q cells will survive radiation therapy than P cells. Therefore, control of Q cells has a great impact on the outcome of cancer therapy. The frequency of closely-spaced DNA damage forming clusters of DNA damage produced by high LET radiation was reported to be less dependent on oxygenation status at irradiation than that of DNA damage produced by low LET γ-ray irradiation [22]. Therefore, for both total and Q tumor cells, neutrons only irradiation was less dependent on oxygenation and had higher RBE values for Q cells than total tumor cells (Table 1) [22]. Neutrons only radiation therapy is thought to be a promising treatment modality for refractory tumors in terms of overall tumor cell-killing effects, including intratumor Q cell control.

The Q tumor cells have been shown to have a much larger hypoxic fraction than the total tumor cells, and hypoxic cells are believed to have less uptake than aerobic cells [6]. Thus, the distribution of ^10^B from the ^10^B delivery agent to Q tumor cells, is less dependent on cell uptake capacity than drug diffusion. Furthermore, considering that the cellular distribution of ^10^B from BSH is highly dependent on drug diffusion, whereas that of ^10^B from BPA is dependent on the uptake ability of tumor cells, the value of the CBE factor for BPA was higher than BSH in the total tumors, but in the Q tumor cells that for BPA was lower than BSH. Thus, combination with BPA and BSH may be one of the promising techniques in BNCT [6, 23]. On the other hand, in terms of cell survival, SAS/*mp53* tumor cells showed higher CBE and RBE values because they were more resistant to γ-ray irradiation than SAS/neo tumor cells. However, at the net MN frequency, SAS/neo and Q tumor cells had higher CBE and RBE values, because SAS/*neo* and Q tumor cells originally showed smaller net MN frequencies to γ-ray irradiation than SAS/*mp53* and total tumor cells, respectively [18, 24].

Meanwhile, the increase in ^10^B concentration in the tumor did not catch up with the increase in the concentration of administered ^10^B delivery agents. That is, when BPA was used, the CBE factor value tended to decrease as the concentration of ^10^B delivery agent administered increased, especially in the Q and SAS/*neo* tumor cells. The decrease in the CBE value indicates that uniform distribution of ^10^B across solid tumors, particularly Q and SAS/neo tumor cells, is difficult, partly due to the effect of tumor heterogeneity. Thus, this may mean that the CBE value is thought to reflect the level of intratumor heterogeneity in solid tumors [2, 6].

Even in BNCT, Q tumor cells are less radio-sensitive than the total tumor cells when ^10^B delivery agents, especially BPA are used, or when *TP53-wild type* SAS/neo tumors are treated, compared to neutron beam irradiation alone (Table 3) [6]. Therefore, more Q tumor cells can survive BNCT than P tumor cells. Furthermore, when ^10^B delivery agents were used, the difference in radio-sensitivity between total and Q tumor cells increased as the concentration of ^10^B delivery agent administered increased. This was partly because the increased distribution of ^10^B from the ^10^B delivery agents to the Q tumor cells did not keep up with the increased concentration of administered ^10^B delivery agents. The heterogeneity of the microenvironment within the tumor has a greater effect on the ^10^B concentration in solid tumors especially when higher concentrations of ^10^B delivery agent are administered. The newly developed ^10^B-containing compound has to be not only non-toxic to normal cells, but also ^10^B from the drug has to be delivered as uniformly as possible throughout the tumor [6].

SAS/*neo* solid tumors exhibited greater ^10^B uptake capacity than SAS/*mp53* solid tumors after administration of ^10^B delivery agents [18]. This was thought to be partly due to the higher cellularity observed in SAS/*neo* tumors than in SAS/*mp53* tumors [24]. This may be also partly because cells containing dominant-negative *p53* proteins escape from the checkpoint mechanism for maintaining genomic stability and proliferate without cell cycle arrest and apoptosis, affecting cell proliferation and ^10^B uptake capacity [3, 4].

Therefore, in terms of the cell survival, the difference in radio-sensitivity between SAS/neo and SAS/*mp53* tumors was more increased and the values of the DMF factors became larger, especially when BPA was used, than when no ^10^B delivery agent was used. However, this increase is rather suppressed as the amount of the administered ^10^B delivery agent increased [18]. In terms of the net MN frequency in total tumor cells as a whole, since more ^10^B was distributed in SAS/*neo* tumors than in SAS/*mp53* tumors when a ^10^B-carrier was used, the difference in the radio-sensitivity between SAS/*neo* and SAS/*mp53* tumors was decreased and the values of the DMF factors became smaller than when no ^10^B delivery agent was used. Namely, even when the ^10^B delivery agent was used, the value of net MN frequencies itself was still larger in SAS/*mp53* than in SAS/*neo*, thus the values of the DMF factors became smaller, and this degree of reduction is rather suppressed as the amount of the administered ^10^B delivery agent increased. Meanwhile, in Q tumor cells, the overall change in the values of the DMF factors in Q tumor cells is less pronounced than in total tumor cells although the tendency of the change was almost similar to that in total tumor cells. In other words, the difference in the characteristics in Q tumor cells between SAS/*neo* and SAS/*mp53* tumors is not as remarkable as in total tumor cells [24]. Both in terms of the cell survival and the net MN frequency, the use of BSH instead of BPA as a ^10^B delivery agent mitigated all changes in the values of the DMF factors [18].

In current clinical BNCT, BPA is always used as a ^10^B delivery agent with or without BSH [25]. Based on the findings of this study, the distribution of ^10^B from BPA may be more affected by heterogeneity in tumors than that from BSH, especially when the tumor is composed of *TP53 wild-type* tumor cells. Thus, the finding that the ability of *TP53 mutant* tumor cells to uptake ^10^B is less dependent on tumor heterogeneity than *TP53 wild-type* tumor cells supports that BNCT has great promise for controlling *TP53 mutant* tumors that are resistant to low-LET radiation. [18]. In fact, the difference in radio-sensitivity between total and Q tumor cells after BNCT was smaller in *TP53 mutant* SAS/*mp53* tumors than in *TP53 wild-type* SAS/*neo* tumors.

Tumor heterogeneity is currently considered to be one of the major difficulties in treating solid tumors. Many tumors contain phenotypically and functionally heterogeneous cancer cells. The established mechanisms involve essential differences between cancer cells caused by stochastic genetic or epigenetic changes (clonal evolution). Differences between cancer cells can also occur through extrinsic mechanisms by which different microenvironments within tumors give cancer cells in different locations different phenotypes and functions [26]. It is well known that tumor blood vessels are characteristically dilated, saccular and tortuous, with large endothelial junctions, increased numbers of fenestrations and lack of normal basement membrane. The abnormal structure of tumor blood vessels impairs blood flow and interferes with effective convective fluid transport, resulting in impaired distribution of blood-borne therapeutics [20, 21]. Therefore, increasing the dose of the ^10^B compound may not be effective however much amount of agent is administered. Recently, a positive association between cancer stemness and multiple measures of intratumoral heterogeneity across cancer was reported [26]. Intratumor heterogeneity may contribute to cancer stemness by both enhancing the replication capacity of individual tumor clones and protecting antigenic clones from elimination by the immune system. This means that cancers are positively correlated with intratumor heterogeneity, which is more likely to be observed and recognized for potential mechanisms of immunosuppression associated with cancer stemness. [26, 27]. Now, heat treatment (hyperthermia) activates antitumor immunity through releasing tumor antigens by extracellular heat shock proteins. Heat-induced antitumor immunity may lead to contribute to the control of tumor recurrence and metastasis [28].

The CBE factor for each tissue and tumor, that was shown to be highly dependent on the degree of ability of distributing ^10^B from ^10^B delivery agents, may be one of the promising candidates as the index to estimate tumor heterogeneity. In the future, we would like to analyze the CBE factor in detail not only as a parameter of BNCT but also as one of the important indices for assessing intratumor heterogeneity.
